# Mink (*Neovison vison*) kits with pre-weaning diarrhea have elevated serum amyloid A levels and intestinal pathomorphological similarities with New Neonatal Porcine Diarrhea Syndrome

**DOI:** 10.1186/s13028-018-0403-7

**Published:** 2018-08-15

**Authors:** Ronja Mathiesen, Julie Melsted Birch, Mariann Chriél, Henrik Elvang Jensen, Jens Frederik Agger, Peter Mikael Helweg Heegaard, Tina Struve

**Affiliations:** 1 Innate Immunology Group, National Veterinary Institute, Technical University of Denmark, Kemitorvet, Building 204, 2800 Kgs. Lyngby, Denmark; 2 Present Address: Innate Immunology Group, Department of Biotechnology and Biomedicine, Technical University of Denmark, Kemitorvet, Building 204, 2800 Kgs. Lyngby, Denmark; 3 Department of Veterinary and Animals Sciences, Faculty of Health and Medical Sciences, University of Copenhagen, Ridebanevej 3, 1870 Frederiksberg C, Denmark; 4 Diagnostics & Scientific Advice, National Veterinary Institute, Technical University of Denmark, Kemitorvet, Building 204, 2800 Kgs. Lyngby, Denmark; 5 Kopenhagen Fur, Langagervej 60, 2600 Glostrup, Denmark

**Keywords:** Bacteriology, Histology, Mink kits, (*Neovison vison*), Pre-weaning diarrhea, Serum amyloid A

## Abstract

**Background:**

Pre-weaning diarrhea (PWD) is a syndrome affecting farm-raised neonatal mink kits. Apart from diarrhea it causes greasy skin exudation, dehydration, and distressed behavior and can ultimately lead to death. No specific causative agents have been identified and the syndrome is regarded as multifactorial. The aim of the present study was to investigate a possible inflammatory state in mink kits with PWD, as indicated by raised serum concentrations of the acute phase protein serum amyloid A (SAA) and by changes in intestinal pathomorphology and intestinal contents of bacteria. Samples collected from 20 diarrheic mink kits with PWD and 20 age-matched non-diarrheic control mink kits from two commercial Danish farms during the pre-weaning period (April–May) in 2016 were analyzed.

**Results:**

Concentrations of SAA in serum samples from mink kits with PWD were significantly higher (up to 1000-fold) compared to non-diarrheic control mink kits. Significant features of enterocytic vacuolization, atrophy and fusion of villi in jejunum and mucosal atrophy of the colon of kits with PWD were found. Moreover, attachment of coccoid bacteria to enterocytes was more often found in kits suffering from PWD, while intra-cytoplasmic eosinophil bodies were more frequently observed in control kits. Cellular infiltrations with mononuclear and neutrophil leukocytes were not associated with disease status. Bacteria from the *Staphylococcus intermedius* group, such as *Staphylococcus delphini,* were more frequently cultivated from control mink kits, whereas *Enterococcus* spp. dominated in mink kits with PWD. *Escherichia coli* was cultivated from both control and mink kits with PWD, but with a higher frequency from mink kits with PWD.

**Conclusion:**

A significant increase in circulating concentrations of SAA was found in PWD affected mink kits from 6 to 23 days old compared to controls. The histopathological changes in PWD mink kits suggest that the type of diarrhea is secretory. Attachment of coccoid bacteria, therefore, might be responsible for an enterotoxic effect causing a loss of balance in movements of ions and water leading to the vacuolization and swelling of the enterocytes. The slight to moderate infiltrations of neutrophils irrespectively of diarrheic status and the attachment of coccoid bacteria to enterocytes are comparable to observations found in piglets suffering from New Neonatal Porcine Diarrhea Syndrome. Mechanisms behind the correlation between increased SAA levels and the observed pathological intestinal features remain obscure.

## Background

The pre-weaning diarrhea syndrome (PWD) in mink (*Neovison vison*) kits is a major cause of concern in the mink industry due to both economic losses and decreased animal welfare. It may affect more than 30% of the litters [1, 2] and has been observed in farm-raised neonatal mink for several decades worldwide [3]. Mink kits affected by PWD display diarrhea with concomitant excessive secretions from the cervical apocrine glands, and exudate on the skin surface, the tail, and the claws. Moreover, dehydration may ultimately lead to the death of the affected kits [4, 5]. The onset of clinical signs generally occurs in the whole litter during the pre-weaning period, at 5–20 days of age, with a morbidity rate varying from 0 to more than 30% of the litters, and a mortality of typically one or two kits per litter [6, own findings, 2017]. The PWD syndrome is considered multifactorial, and there is a lack of consistency in isolated bacteria and viruses in kits with PWD. Studies have aimed to define the causality of the syndrome and mink astrovirus (MiAstV) isolated from mink kits with PWD indicate involvement in the syndrome [7–11]. However, all of the proposed putative pathogens including also *Campylobacter jejuni*, rota-, calici-, and mink coronavirus, *Escherichia coli*, and *Staphylococcus delphini* have also been isolated from clinically healthy kits, so their role in PWD remains elusive [10–13]. Apart from having a multifactorial infectious origin, other factors including management factors have been associated with an increased risk of developing PWD. For example, litters from 1-year old females and in females with low energy supply in the late gestation period are at increased risk of being affected by PWD [2, 14]. Moreover, the presence of dogs on the farm area as well as the size of the farm (total number of females) have also been associated with high morbidity of PWD [2]. Regarding the intestinal pathomorphology accompanying PWD, only a few studies have been published [15, 16]. Moreover, intestinal lesions in mink kits suffering from PWD have not been classified, according to the standard pathomorphological paradigm, as either non-inflammatory/secretory, inflammatory or invasive [17]. A possible biomarker to assess infection or inflammation in mink kits suffering from PWD is serum amyloid A (SAA). SAA is an acute phase protein found in low concentrations in healthy animals and is released following inflammation, infection, or tissue injury in both mink [18–20] and many other species, including humans [21, 22]. It is synthesized predominantly by the liver in response to the cytokine interleukin 1, however, other organs such as the intestine, have also been shown to produce it [19]. The aim of the present study was to examine if the levels of SAA could be a biomarker for PWD in mink kits and to characterize and compare the intestinal pathomorphology, and the bacterial intestinal contents between healthy controls and mink kits suffering from PWD.

## Methods

### Animals

In total, 20 mink (*Neovison vison*) kits with PWD and 20 age-matched healthy kits (controls), between 6 and 23 days old, were obtained during the pre-weaning period (April–May) from two (A and B) commercial Danish mink farms with outbreaks of PWD. Sick mink kits were selected based on the liquid consistency of the feces and the following clinical manifestations: sticky cutaneous exudation, red swollen anus and perineal soiling (own findings, 2017). Mink female and their kits were housed in separate cages with conventional nest boxes, females were fed conventional feed and had unlimited access to water. Mink kits were euthanized with CO or CO_2_, bled, and the intestine was removed aseptically immediately thereafter.

### Serum amyloid A ELISA

Un-stabilized blood samples from the kits were obtained at the time of euthanasia. Blood was allowed to clot and serum was obtained as the supernatant after centrifugation at 4000*g* for 15 min at 4 °C. Serum was stored at − 20 °C until analysis. A commercially available multispecies sandwich ELISA (Phase SAA assay, Tridelta Development Ltd., Kildare, Ireland, #TP 802) was used to quantify SAA concentrations in the serum samples. This assay is a quantitative sandwich ELISA using rat anti–human monoclonal antibodies [23]. Mink kit serum samples were diluted 500 times according to the manufacturer’s instructions for canine SAA. The lower limit of quantification was 6.25 μg/mL (canine SAA units) as given by the lowest concentration of the standard dilution series included on each plate. Readings below the lower limit of quantification were assigned the value 6.3 µg/mL. Data was transferred to GraphPad Prism version 7 (GraphPad Software, San Diego, California, USA, https://www.graphpad.com) for graphic representation and for statistical analysis. Significant difference in SAA concentrations in circulation between PWD kits and control kits were identified using the Mann–Whitney test in Prism. P values < 0.05 were considered significant.

### Histology

Duodenal, mid-intestinal (jejunum) and aboral colon sections from each kit were fixed with needles on a piece of styrene foam and placed in 10% neutral buffered formalin in a CellStor Pot (CellPath, Newtown, Powys, UK). Two pieces from each of the three gut sections were embedded in paraffin and sections of 4–5 µm were prepared and stained with hematoxylin and eosin (HE). Selected sections were stained with Periodic acid–Schiff (PAS), Gram, and Warthin–Starry (WS). Each of the three sections was histologically examined and graded blindly according to the following manifestations (Table 1).

**Table 1:** Histological criteria for assessing sections from duodenum, jejunum and aboral colon of mink kits

Histological examinations	Assessment	Score
Degree of vacuolization of the enterocytes	Loss of staining of the cytoplasm in a group of enterocytes seen together with elongation and hypertrophy due to micro-vacuolization	Absent (0) Mild: present focally (1) Severe: present multifocal or disseminated (2)
Coccoid bacteria and rod-shaped bacteria (duodenum and jejunum)	Attached to enterocytes on the villi	Absent (−) Present (+)
Semi-quantified degree of infiltration of neutrophils (duodenum and jejunum)	Neutrophils in villi	≤ 5 neutrophils/villi (0) Several villi contained 5–10 neutrophils (1) Several villi contained > 10 neutrophils (2)
Infiltrations of mononuclear cells	Distinguished from Peyer’s patches as diffuse, disorderly infiltrations of mononuclear cells in the lamina propria	Absent (−) Present (+)
Intra-cytoplasmic eosinophil bodies of the enterocytes	Circular elements often located adjacent to the nuclei [15, 16]	Absent (−) Present (+)
Atrophy and fusion of the villi (duodenum and jejunum)	Reduction of the height of villi and the spaces between them [15]	Absent (−) Present (+)
Amount of enterocytic proliferation (duodenum and jejunum)	Number of mitosis in enterocytes	Number
Atrophy of mucosal lining (colon)	Reduction of mucosal height and normal architecture	Absent (−) Present (+)

For each of the included animals a score for duodenum + jejunum and colon sections was established, respectively. Thus, for the dichotomous variables the summary score was “absent” (−), if absent in all gut sections and “present” (+), if present in any of the gut sections. For the assessment of vacuolization a cumulated score was established and categorized as absent (−) if the cumulated score was 0, or present (+) if the cumulated score was > 0. For the assessment of infiltration with neutrophils in the villi a cumulated score was established, and categorized based on the mean score as high or low i.e. ≥ 2/< 2 for duodenum + jejunum sections. Associations between disease status of the kit and each variable was tested with the SAS Enterprise Guide 7.1 using Chi square and where relevant Fisher’s exact test (expected < 5). A cumulated number of mitosis in enterocytes of duodenum and jejunum from PWD and control mink kits were tested with Students t-test. P values < 0.05 were considered significant.

### Bacteriology

Post mortem, the intestine was removed from the abdomen as previously described [24]. The aboral part of the colon was aseptically opened with a sterile scalpel and swabbed for bacterial cultivation (Transport Swabs, VWR, Radnor, PA, USA). The total of 17 pooled swab control samples were generated by pooling swabs from two healthy kits from the same litter, on the same day, from 17 litters age-matched to the kits affected by PWD. The age-matching of the mink kits unfortunately resulted in some of the kits being pooled into the same tube. This left only 17 pooled samples, instead of 20, from kits without PWD. From the diarrheic kits, the samples were cultured individually. The swabs were stored at 5 °C until analysis by blinded cultivation. The swabs were inoculated on blood agar plates enriched with 5% bovine blood (blood agar base, Oxoid, Thermo Scientific, Watham, MA, USA) and incubated at 37 °C aerobically for 1–2 days. The two most frequently occurring bacterial colonies were then sub-cultivated before they were identified by Matrix-Assisted Laser Desorption Ionization Time-Of-Flight mass spectrometry (MALDI–TOF–MS) using a VITEK MS MALDI-TOF (BioMérieux, Marcy-l’Etoile, France) as previously described [25]. If two bacterial species were equally frequent next to a single more frequent species both were sub-cultivated, and hence three isolates were identified.

## Results

### Serum SAA concentrations

**Fig. 1:**
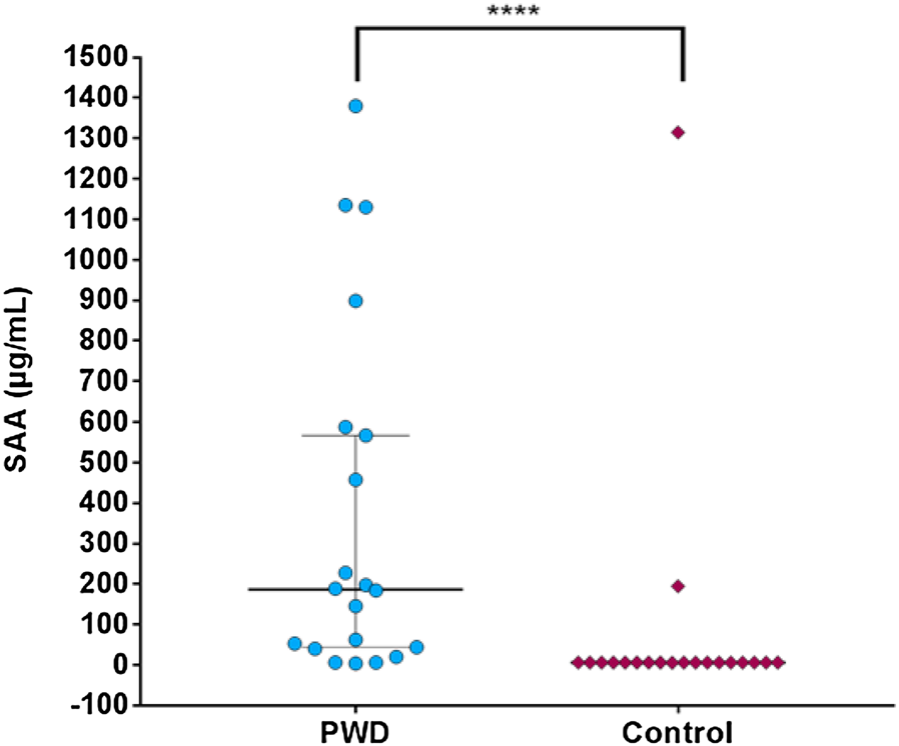
Serum concentration of serum amyloid A (SAA) protein in PWD and healthy control mink kits as determined by SAA ELISA. Median of the serum SAA concentrations with 95% confidence interval is depicted (n = 20). The Mann–Whitney test showed a statistically significant higher concentration of serum SAA in PWD kits than in control kits (****P < 0.0001)

The difference in serum SAA concentration between PWD kits and control kits was highly statistically significant (Mann–Whitney test, P < 0.0001) (Fig. 1). Only 2/20 (10%) control mink kits had SAA concentrations higher than the lower limit of quantification of 6.3 µg/mL, while 17/20 (85%) kits with PWD had SAA concentrations higher than 6.3 µg/mL.

### Histology

**Fig. 2:**
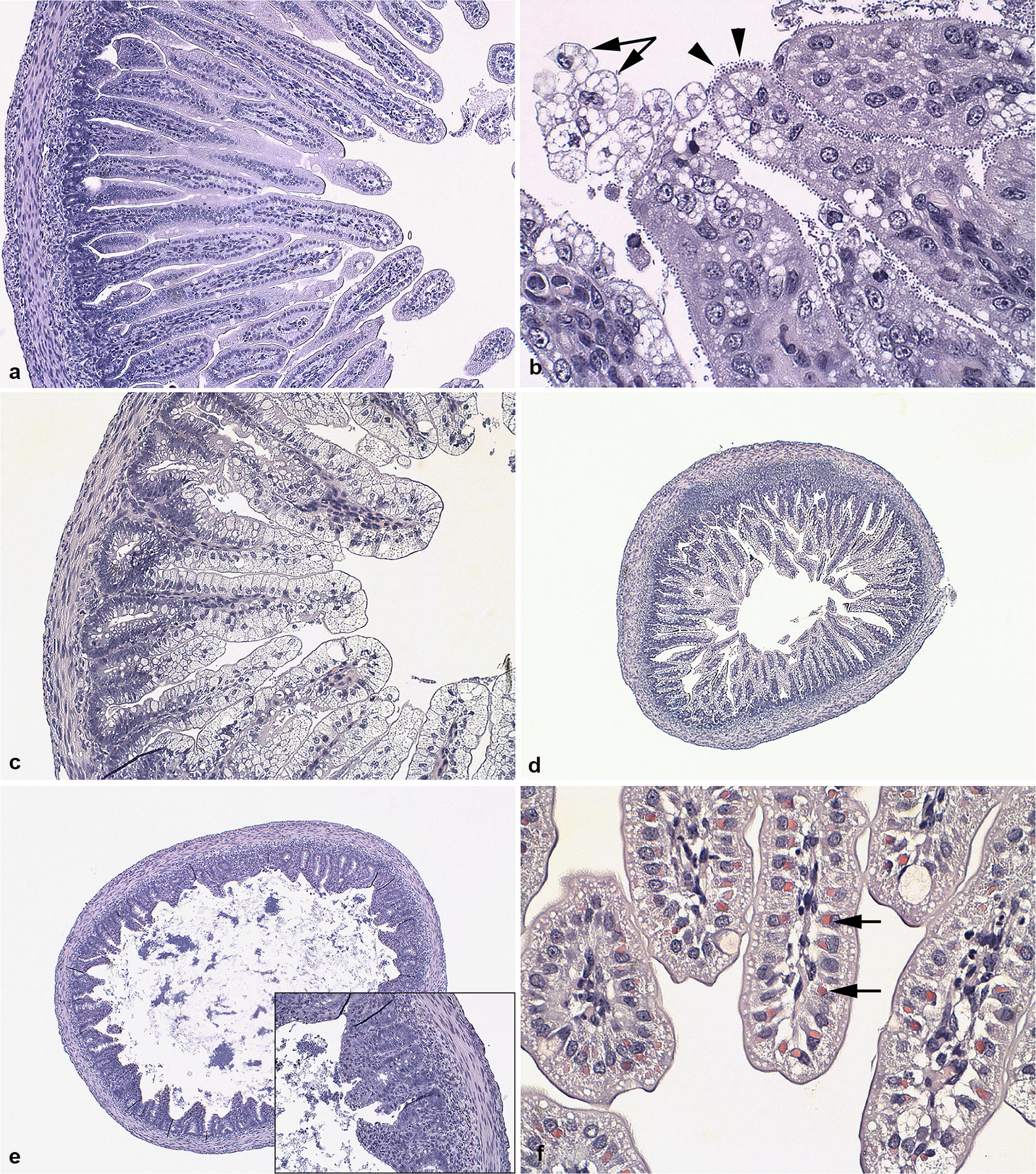
Photomicrographs of representative examples of intestines from mink kits. **a** Jejunum from healthy mink kit. **b** Vacuolization on the tip of the villi (arrows) and pronounced attachment of coccoid bacteria (arrow heads) to the enterocytes in jejunum of a mink kit with PWD. **c** Severe vacuolization and hypertrophied enterocytes in the colon of a mink kits with PWD. **d** Colon from a healthy mink kit. **e** Atrophy of the mucosa of the colon from a mink kit with PWD. Inset: higher magnification of the mucosal atrophy. **f** Eosinophilic bodies in the enterocytes from jejunum in a healthy mink kit (arrows)

The results of the histologic evaluations are presented in Table 2. Compared to normal kits (Fig. 2a) PWD mink kits expressed significant features of enterocytic hydropic vacuolization (PAS negative) in the jejunum and colon (Fig. 2b, c). Atrophy and fusion of villi in the small intestine and atrophy of the mucosa lining in the colon were also associated with presence of the disease, and were most prominent in jejunum and colon (Fig. 2d, e). The association between attachment of coccoid bacteria to enterocytes and disease status was highly significant in duodenum + jejunum and colon (P < 0.0001 and P = 0.001, respectively), and was equally present in all parts of the intestine (Fig. 2b). Selected Gram stained sections revealed that the coccoid bacteria were Gram positive. Rod-shaped bacteria were also significantly observed more frequently in intestines from PWD kits, but the attachment to villi was sporadic and in combination with coccoid bacteria. Intra-cytoplasmic eosinophilic bodies (PAS positively stained vacuoles) were significantly more frequent in control kits compared to PWD kits, and most often in jejunum (Fig. 2f). Infiltrations with mononuclear and neutrophil leucocytes were not associated with disease status. Moreover, the mitotic activity of enterocytes in the small intestine were significantly higher in PWD kits compared to control kits (P = 0.003).

**Table 2:** Histopathological findings in duodenum ± jejunum and colon from control to PWD mink kits, respectively

Histopathologic features	PWD (n = 20)	Control (n = 20)	P-value
Duodenum + jejunum			
Vacuolization (hydropic degeneration) of enterocytes (+/−)	8/12	1/19	0.02
Coccoid bacteria attached to enterocytes (+/−)	16/4	1/19	< 0.0001
Rod-shaped bacteria attached to enterocytes (+/−)	4/16	0/20	0.11
Infiltration of neutrophils in villi (high/low)	9/11	14/6	0.11
Infiltration of mononuclear cells (+/−)	0/20	0/20	–
Eosinophilic bodies in enterocytes (+/−)	2/18	12/8	0.001
Atrophy of villi (+/−)	4/16	0/20	0.11
Fusion of villi (+/−)	5/15	0/20	0.05
Number of mitosis [mean, (standard deviation)]	66.3 (30.3)	39.8 (20.9)	0.003
Colon			
Vacuolization (hydropic degeneration) of enterocytes (+/−)	11/9	1/19	0.001
Infiltration of mononuclear cells (+/−)	3/17	1/19	0.61
Eosinophilic bodies in enterocytes (+/−)	2/18	4/16	0.66
Atrophy of the mucosa (+/−)	9/11	1/19	0.01

### Bacteriology

The number of bacterial isolates obtained from healthy mink kits and kits with PWD is shown in Table 3. The dominating species cultivated from healthy control mink kits was from the *Staphylococcus intermedius* group, whereas *Enterococcus* spp. was the main species found in mink kits with PWD. On Farm B, *E. coli* was cultivated more frequently from mink kits with PWD (58.8% of the isolates) than from control kits (41.7% of isolates). On farm A, *E. coli* was not isolated from any of the control kits, but accounted for 28.6% of the isolates from the mink kits suffering from PWD.

**Table 3:** Number and percentages of bacterial isolates from control and PWD mink kits

Bacterial isolates	Farm A		Farm B	
	Control^a^	PWD^b^	Control^a^	PWD^b^
Number of samples	9	10	8	10
*Escherichia coli*	–	4 (28.6%)	5 (41.7%)	10 (58.8%)
*Staphylococcus* *intermedius* group	7 (58.3%)	2 (14.3%)	6 (50%)	1 (5.9%)
*Staphylococcus schleiferi*	2 (16.7%)	–	–	–
*Enterococcus faecalis*	1 (8.3%)	–	–	–
*Enterococcus faecium*	2 (16.7%)	8 (57.1%)	–	–
*Enterococcus durans*	–	–	1 (8.3%)	–
*Enterococcus hirae*	–	–	–	6 (35.3%)
Total number of isolates	12	14	12	17

^a^The samples from the control kits from each farm were pooled samples (two kits from the same litter, sampled on the same day, constituted the samples)

^b^The samples from the PWD kits were not pooled

## Discussion

We report for the first time that PWD in mink kits between 6 and 23 days old is associated with a significant increase in circulating concentrations of SAA. Two of the control kits showed elevated concentrations of SAA in serum, however only one of them (1315 µg/mL; Fig. 1) showed signs of cholangitis, which could explain the elevated concentration of SAA (data not shown). SAA have been shown by Bruun et al. [20] to be elevated in mink after subcutaneous injection with lipopolysaccharide from *E. coli*. The gastrointestinal tract of mink is very short and the passage of feed through the entire gastrointestinal tract takes approximately 2–3 h [26]. Previous studies have shown the difficulty in culturing bacteria from the small intestine in mink [27] and therefore we decided to use swabs from the colon, where the density of bacteria is relatively high. Furthermore, the decision to only cultivate the bacteria aerobically was based on the fact that only aerobic bacteria have so far been isolated from PWD-affected mink, whereas anaerobic bacteria have not [11, 28, 29]. We isolated *E. coli* most frequently from kits suffering from PWD, which corresponds well with previous findings that *E. coli* is commonly isolated from PWD mink kits [11, 16, 30, 31]. A quantitative study of healthy mink kits showed that the intestinal counts of *E. coli* were highest when kits were around 4 weeks of age [30], but in the present study *E. coli* was isolated from younger kits suffering from PWD. However, *E. coli* has also been identified as part of the normal mink kit intestinal microflora [11, 16, 30, 32]. Moreover, analysis of virulence factors of *E. coli* has revealed that the population of *E. coli* in mink consists of several serogroups with no apparent association to outbreaks of PWD [11, 32]. In general, enterococci are not regarded as pathogenic in mink, but *Enterococcus hirae* has been associated with diarrhea in 4–7 week old mink kits submitted for diagnostic testing (Chriél, pers. comm., 2017), as well as in other neonatal animals such as suckling rats, kittens and piglets [33–35]. Gut sections from PWD mink kits showed clear disease associated changes, including vacuolization of enterocytes and pronounced attachment of coccoid bacteria, both of which have been observed in other studies [7, 15, 16]. The intracytoplasmic eosinophilic bodies (PAS positive vacuoles) within the enterocytes that most frequently were identified in the healthy mink kits have also been described previously [15, 16]. Although their role has not been clarified in mink, their staining properties and localization is comparable to the absorptive, intra-cytoplasmic vacuoles found in neonates of pigs and ruminants [36]. Therefore, the presence of these vacuoles in enterocytes of healthy mink kits is not surprising and may be related to the intestinal uptake of maternal milk antibodies [37], taking place up to 4–5 weeks after birth in mink kits [37]. Although more pronounced changes of the gut architecture like atrophy and fusion of villi were present in the PWD kits, no significant difference in the degree of neutrophil and mononuclear leucocyte infiltration were observed between controls and PWD mink kits. This lack of histopathological signs of inflammation indicates that PWD in the mink kits represents a secretory type of diarrhea. The observed attachment of coccoid bacteria may be responsible for an enterotoxic effect causing a loss of balance of movements of ions and water leading to the vacuolization and swelling of the enterocytes. Interestingly, the attachment of enterococci and *E. coli* to enterocytes and the slight to moderate infiltrations of neutrophils irrespective of diarrheic status has recently been found in piglets suffering from New Neonatal Porcine Diarrhea Syndrome (NNPDS) [38–40], suggesting similarities in mechanisms between diarrhea in the pre-weaning period of mink kits and piglets. Elevated levels of SAA and adhesion of bacteria to the intestinal wall has been seen for segmented filamentous bacteria (SFB), which adhere to the enterocytes, inducing epithelial SAA production [41, 42]. Atarashi et al. [43] colonized rats and germ-free mice with SFB from 20 strains of bacteria isolated from feces from patients suffering from ulcerating colitis and from *E. coli* and found that they all promote the induction of Th17 cells, which in turn could lead to an increased SAA production in enterocytes. Research on local expression of SAA in the intestine is needed to elucidate if circulating SAA levels are increased as a consequence of local production of SAA by epithelial intestinal cells in mink kits affected by PWD. In rodent models, a local induction of SAA in enteric epithelial cells in response to an altered microbiota in the absence of inflammation has indeed been demonstrated [43], however the impact on circulating SAA concentrations has not been reported. Although more pronounced changes of the gut architecture, like atrophy and fusion of villi were present in the PWD kits, no significant difference in the degree of neutrophil and mononuclear leucocyte infiltration were observed between controls and PWD mink kits. Thus, the possibility of an association between increased SAA levels and an unidentified inflammatory state in mink kits suffering from PWD should be investigated in more detail.

The most frequent bacterial isolate from control mink kits belonged to the *S. intermedius* group, which in mink has been shown to be *S. delphini* [44]. This finding is in line with previous studies of the normal intestinal microflora of mink kits [12, 30]. Although mink are natural hosts of *S. delphini* this bacterial species was suggested to be responsible for an outbreak of PWD through enterotoxin production, which again leads to the question if *S. delphini* could be an opportunistic bacterium after a primary infection during an episode of PWD [29]. In contrast, MiAstV, mink coronavirus, mink enteritis virus, and rotavirus A were not found by PCR [45] in any of the kits analyzed (data not shown), suggesting a low presence of virus.

## Conclusions

We identified a significant increase in circulating concentrations of SAA and attachment of coccoid bacteria in kits affected by PWD. The slight to moderate infiltrations of neutrophils irrespectively of diarrheic status and the attachment of coccoid bacteria to enterocytes show similarities with observations found in piglets suffering from NNPDS, and suggest that PWD in mink is a secretory type of diarrhea.
